# Towards a Personalized Definition of Prognosis in Philadelphia-Negative Myeloproliferative Neoplasms

**DOI:** 10.1007/s11899-022-00672-6

**Published:** 2022-09-01

**Authors:** Barbara Mora, Francesco Passamonti

**Affiliations:** 1 Hematology, Ospedale Di Circolo, A.S.S.T. Sette Laghi, Viale Borri 57, 21100 Varese, Italy; 2 Department of Medicine and Surgery, University of Insubria, Via Guicciardini 9, 21100 Varese, Italy

**Keywords:** Essential thrombocythemia, Polycythemia vera, Myelofibrosis, Prognosis, Next-generation sequencing

## Abstract

**Purpose of Review:**

Philadelphia-negative myeloproliferative neoplasms (MPNs) include polycythemia vera (PV), essential thrombocythemia (ET), prefibrotic (pre-), and overt-primary myelofibrosis (primary MF, PMF). PV and ET could evolve into secondary MF (SMF), whose early diagnosis relies on monitoring signs of possible progression. All MPNs have a risk of blast phase (BP), that is associated with a very dismal outcome. Overall survival (OS) is different among MPNs, and disease-specific prognostic scores should be applied for a correct clinical management. In this review, an overview of current prognostic scores in MPNs will be provided.

**Recent Findings:**

The biological complexity of MPNs and its role on the trajectory of disease outcome have led to the design of integrated prognostic models that are nowadays of common use in PMF patients. As for PV and ET, splicing gene mutations could have a detrimental role, but with the limit of the not routinary recommended application of extensive molecular analysis in these diseases. SMF is recognized as a distinct entity compared to PMF, and OS estimates should be calculated by the MYSEC-PM (*Myelofibrosis SECondary-prognostic model*). Both in PMF and SMF, decisions as selection of patients potentially candidates to allogenic stem cell transplant or that could benefit from an early shift from standard treatment are based not only on conventional prognostic scores, but also on multivariable algorithms.

**Summary:**

The expanding landscape of risk prediction for OS, evolution to BP, and SMF progression from PV/ET informs personalized approach to the management of patients affected by MPNs.

## Introduction

Philadelphia-negative myeloproliferative neoplasms (MPNs) are clonal hematopoietic neoplasms characterized by the hyper-activation of signal transduction pathways, such as JAK-STAT [1••, 2]. This leads to a pro-inflammatory state and to the overproduction of myeloid blood cells [1••, 2]. In the 2016 *World Health Organization* (WHO) classification, MPNs include polycythemia vera (PV), essential thrombocythemia (ET), prefibrotic (pre-), and overt-primary myelofibrosis (PMF) [1••]. At a median time to progression (TTP) of around 11 years, 10–20% of PV and ET cases evolve respectively into post-PV (PPV-) and post-ET (PET-) myelofibrosis (MF), also known as secondary MF (SMF) [3, 4]. In addition, MPNs have a propensity to evolve in secondary acute myeloid leukemia, also called blast phase (BP), and finally associated to a dismal outcome [5, 6].

MPNs are rare diseases, with an incidence that varies from 0.1 to 2.8/100,000 patients per year in Europe and it is about 0.44/100,000 patients per year in the USA by a recent *Surveillance, epidemiology, and end results* (SEER) report [7, 8]. PV is the most prevalent, while MF the less frequent [7]. These are primarily diseases of adult life, with a median age at onset in the sixth decade [7, 8]. Noteworthy, around 20% of patients are younger [7].

Median overall survival (OS) in ET patients is around 20 years [5, 9], having ruled out a possible differential diagnosis of pre-PMF [1••, 10]. As for PV, median OS was shown to be further reduced compared to an age- and sex-matched US population and equal to 12–14 years after diagnosis [7, 11]. Patients affected by MF have the worst outcome, with a median OS of around 6 and 9 years in PMF and SMF, respectively [12, 13••]. In the SEER program, over 20,000 patients with PV, ET, and PMF have received a diagnosis between 2001 and 2016 in the USA [14]. The 10-year cumulative mortality of PV, ET, and PMF was 18.3%, 12.5%, and 48.5% for patients younger than 60 years and 46.7%, 44.7%, and 83.7% for those above this age cut-off, respectively [14]. Nonclonal progression accounted for less than 10% of cause-specific deaths in PV and ET, but for around one third in PMF cases [14].

Nevertheless, outcome of MF patients is improving in the last years [15•, 16•], thanks to earlier diagnosis [1••], increased knowledge of the genetic background [17], introduction of JAK inhibitors, and a greater experience with selecting and managing allogeneic hematopoietic stem cell transplant (allo-SCT) [18••, 19•, 20••, 21].

The risk of BP transformation in MPNs depends on the subtype: in the first decade of the disease, it is equal to 10–20%, 3%, and less than 1% in PMF, PV, and ET, respectively [9].

Conventional prognostic models in MPNs are based on “day-to-day practice” parameters, like demographic data and complete blood count (CBC) values. However, the biological complexity of these diseases, the discoveries related to their molecular landscape, and the increasingly frequent diffusion of *next-generation sequencing* (NGS) methods have led to the design of integrated prognostic models, able to provide a better definition of outcome, especially in MF.

MPNs are characterized by phenotypic driver gene mutations, involved in various ways in the downstream activation of JAK-STAT signaling with consequently different clinical features [1••, 2]. Approximately 95% of PV patients present the *JAK2*V617F mutation in exon 14, with most of the remaining 5% of patients having a *JAK* exon 12 mutation [17]. *JAK2*V617F mutation is observed in around two-thirds of ET and MF patients [17, 22••]. The latter two harbor *CALR* (type 1, 2, or others) and *MPL* alterations in around 30% and 5% of cases, respectively [17, 22••]. Of note, these driver mutations could co-occur in up to 30% of ET and MF cases with low (< 5%) *JAK2*V617F allele burden (AB), with “double mutated” subjects being more often characterized by higher platelets count vs “single mutated” low *JAK2*V617F AB positive cases [23]. Approximately 10% of ET and MF patients do not have any of the canonical three driver mutations and are referred to as “triple-negative” (TN) cases [17]. Additional non-driver myeloid neoplasms-associated gene variants (M-GVs) have been identified in MPNs [17]. Mutations can occur in several classes of genes: epigenetic modifiers (*DNMT3A*, *TET2*, and *ASXL1*), splicing factors (*SF3B1*, *SRSF2*, and *U2AF1*), metabolic enzymes (*IDH1* and *IDH2*), and tumor suppressors (*TP53*) are the most involved [17].

Besides, a study based on over 2000 patients with MPNs (mainly ET) that underwent extensive gene sequencing identified eight different genomic subgroups, combining information on driver and additional mutations [24•]. Chromatin-related, spliceosome or *TP53* mutations have been identified and could impact the outcome also in MPN patients with concomitant splanchnic vein thrombosis (SVT), a group usually represented by young patients [25].

In this work, we will retrace the evolution of prognostic definition in MPNs, with a special attention to the added value given by the information related to their molecular background. As for MF, we will also stress the importance of a correct outcome prediction in SMF cases, in patients potentially candidates to allo-SCT or undergoing ruxolitinib (RUX) therapy.

## Essential Thrombocythemia and Polycythemia Vera

### Predictive Factors of Survival and Blast Phase Evolution

In ET and PV, treatment indications are based on thrombotic risk [26••, 27••]. In a population study of a wide cohort of MPN cases, cardiovascular disease accounted for around 25% of deaths [28]. Besides, on a large number of Medicare beneficiaries, mortality was increased for patients that experienced a thrombosis [29]. It is therefore current practice to tailor patients’ monitoring on their thrombotic risk more than on OS estimates [26••, 27••]. Nevertheless, both conventional and integrated prognostic models have been developed in ET and PV.

The IPSET (*International Prognostic Score for ET*) score considers as risk factors for OS: age at least 60 years (2 points), leukocytes count ≥ 11 × 10^9^/L (1 point), and history of thrombosis (1 point) [30]. Compared to low-risk cases (total score 0, OS not reached), patients classified as intermediate risk (total score 1–2) have a median OS of 24.5 years, while patients identified as high risk (total score 3–4) have 13.8 years [30]. The model works well also in the prediction of thrombosis [30].

Information on driver mutation status does not appear to impact OS in ET [9, 31]. In a recent paper of 809 ET cases, OS was reduced in case of abnormal karyotype (AK), even though it could be found in less than 10% of subjects [32].

Risk factors for BP transformation in ET are various among studies and reviewed in [33]: laboratory parameters (anemia, extreme thrombocytosis, leukocytosis), age, previous thrombosis, bone marrow fibrosis (BMF) grade and cellularity, evidence of cytopenia after hydroxyurea (HU) use [33]. Of note, some of the clinical and morphological predictive characteristics could underline a misdiagnosed pre-PMF case [1••, 10].

In a study of 1545 PV cases by the *International Working Group-Myeloproliferative Neoplasms Research and Treatment* (IWG-MRT), age at least 67 years (5 points), age 57–66 years (2 points), leukocyte count at least 15 × 10^9^/L (1 point), and venous thrombosis (1 point) were combined to devise a prognostic model that distinguished low- (0 points), intermediate- (1–2 points), and high-risk (at least 3 points) categories [11]. The latter (36% of patients) had a median OS of 10.9 years, intermediate (31%) of 18.9 years, and low risk (33%) of 27.8 years [11]. Pruritus was identified as being prognostically favorable [11]. If the IWG-MRT model does not include gender among variables, another retrospective study showed that females are at lower risk of death, leaving this topic open to debate [34].

Almost all PV cases present the *JAK2*V617F mutation; therefore, its AB has been investigated as a possible prognostic marker, but not definitive conclusion could be driven in terms of impact on OS at the moment [31]. In the overmentioned study of 1545 PV patients, there was no difference in OS between patients with *JAK2*V617F vs other *JAK2* mutations [11]. AK, present in around 20% of PV cases, has been correlated with reduced outcome, even though no further cytogenetic sub-classification has been performed to date [35].

Risk factors for BP evolution in PV were identified as older age, AK, leukocytes at least 15 × 10^9^/L, and exposure to old-fashioned cytoreductive treatments like pipobroman or P32/chlorambucil [11, 35–37]. No association was found between BP incidence and HU or busulfan use [11]. Impact of gender on BP transformation is not clear [34, 38].

Recently, the prognostic relevance of additional M-GVs has been investigated in large cohorts of PV and ET patients [39, 40, 41••]. NGS analysis revealed that about half of subjects harbored additional M-GVs, most frequently in *ASXL1* and *TET2* [39]. Prognostically unfavorable M-GVs were present in 15% of cases [39]. In PV, OS was influenced by finding mutations in *ASXL1* and *SRSF2*, while BP-free survival (BP-FS) by *SRSF2* and *IDH2* [39], and in ET, *SH2B3*, *SF3B1*, *U2AF1*, *TP53*, *IDH2*, and *EZH2* [39]*.*

In a retrospective study of 100 *JAK2*V617F-mutated patients with PV or ET, mutations in *ASXL1*, *TP53*, *SRSF2*, *IDH1/2*, and *RUNX1* were associated with BP transformation, reflecting genomic instability [40].

In a collaborative study of the Mayo Clinic and the University of Florence, unfavorable M-GVs were identified in 10% of TE and 2% of PV cases [41••]. Independent genetic risk factors for OS included *SF3B1*/*SRSF2/U2AF1/TP53* mutations in ET and *SRSF2* alterations in PV [41••]. Besides, in ET, *TP53* worsened BP-FS [41••]. These findings led to the incorporation of molecular information into a new integrated prognostic score for PV/ET: the *Mutation-Enhanced International Prognostic Scoring System* for PV (MIPSS-PV) and ET (MIPSS-ET) (Table 1) [41••]. Of note, 63.4% of PV and 50.8% of ET cases are included in the low-risk group, while only 4.4% and 11.6% in the high-risk category [41••]. The predictive ability of these models looks superior to conventional scores [41••]. Therefore, mutations in genes involved in splicing processes and *TP53* seem to have a negative impact in ET and PV. The limited biological activities of current therapies in the market and the not routinary recommended use of NGS methods in the diagnostic process of PV and ET limit the applicability of integrated prognostic scores in the daily practice [26••]. This scenario will change with new compounds with potential disease-modifying properties. Besides, MIPSS-ET/PV models should be validated in large independent cohorts.

**Table 1:** The Mutation-Enhanced International Prognostic Scoring System for polycythemia vera and essential thrombocythemia

	MIPSS-PV	MIPSS-ET
Clinical variables (points)	WBC ≥ 15 × 10^9^/L (1) Thrombosis history (1) Age > 67 years (2)	WBC ≥ 11 × 10^9^/L (1) Male sex (1) Age > 60 years (4)
Molecular variables (points)	*SRSF2* (3)	*SRSF2*, *SF3B1*, *U2AF1*, and *TP53* (2)
OS based on risk (points)		
*Low*	24 years (0–1)	34.3 years (0–1)
*Intermediate*	13.1 years (2–3)	14.1 years (2–5)
*High*	3.2 years (4–7)	7.9 years (6–8)

Legend: *MIPSS-PV*, Mutation-Enhanced International Prognostic Scoring System for polycythemia vera; *MIPSS-ET*, Mutation-Enhanced International Prognostic Scoring System for essential thrombocythemia; *WBC*, white blood cells; *OS*, overall survival

### Predictive Factors of Evolution to Post-polycythemia Vera and Post-essential Thrombocythemia Myelofibrosis

Several studies have investigated the relevance of clinical and molecular features during PV or ET phase, as predictive factors of PPV- and PET-MF evolution [42]. In both diseases, male gender seems associated with higher SMF risk [38]. In the overmentioned retrospective study of 100 patients with PV or ET, mutations in *IDH1/2* or *SF3B1* were associated with SMF-free survival (SMF-FS) [40]. Biological markers of evolution are also under study, as polymorphisms in the chemotactic factor MCP-1 (*monocyte chemoattractant protein-1*) or involvement of the NF-kB (*nuclear factor k-light-chain-enhancer of activated B cells*) signaling [43, 44].

Looking in details at ET cases, it is necessary to make an accurate morphological distinction from pre-MF, since the latter has a higher risk of overt fibrotic evolution [10]. Advanced age, anemia, bone marrow hypercellularity, and BMF grade were correlated to higher probability of progression to PET-MF, although not constantly across all studies [10, 45].

Cytogenetic abnormalities do not seem to predict SMF evolution from ET [32]. In one study, SMF-FS was influenced by *CALR* mutation subtype: *CALR* type 1-like conferred an increased risk of PET-MF, while *CALR* type 2-like was associated with a more indolent course [46]. However, in ET cohorts with a more equal distribution of *CALR* mutations, no specific correlation with SMF-FS has been found [47]. Haider et al. showed a high risk of fibrotic progression in *MPL*-positive ET [48]. Recently, type 1/type 1-like *CALR* and *MPL* were confirmed to be associated with reduced SMF-FS [49]. In the same paper, within *JAK2*V617F positive cases, those with AB > 35% have a higher risk of evolution [49]. As for M-GVs, deep sequencing analysis showed that somatic mutations in at least one gene among *SH2B3*, *SF3B1*, *TP53*, *IDH2*, *EZH2*, and mostly *U2AF1* were associated with shorter SMF-FS [39]. *U2AF1*/*SF3B1* alterations showed a detrimental role in the MIPSS-ET cohort [41••].

As for PV, clinical features with a probable impact on evolution into SMF are leukocytosis and palpable splenomegaly [37, 50]. From a histopathologic point of view, the presence of at least BMF grade 1 and the so-called “megakaryocyte activation” pattern (defined by the coexistence of megakaryocytes emperipolesis, clustering, and surrounding fibrosis) are possible markers of progression [51, 52]. For patients treated with HU, the development of cytopenia and/or the failure to reduce severe splenomegaly could be associated with an increased risk of SMF [53]. In a recent paper that compared PV subjects treated with recombinant interferon (rIFN) vs HU or only phlebotomies, SMF-FS appeared longer with rIFN just for patients classified as low risk for thrombosis [26••, 27••, 54].

Among genetic risk factors, high AB (> 50%) and homozygosity of *JAK2*V617F have been associated with PPV-MF progression [55]. Applying targeted deep sequencing analysis to two different cohorts of PV patients, *ASXL1*, *IDH2*, and particularly *SRSF2* mutations showed an adverse impact on SMF-FS [39]. The role of splicing factor mutations has been confirmed in the MIPSS-PV cohort [41••]. Some chromosomal abnormalities could have a detrimental effect in terms of PPV-MF evolution [35]. Table 2 summarizes the variables associated with increased risk of PET- and PPV-MF.

**Table 2:** Potential risk factors for evolution from polycythemia vera and essential thrombocythemia in secondary myelofibrosis

Variable	ET	PV
Clinical	Male gender Advanced age Distinction from pre-PMF	Male gender Splenomegaly
Complete blood count	Anemia	Leukocytosis
Abnormal karyotype	No	Yes
Bone marrow	Hypercellularity BMF grade	MK activation pattern BMF grade at least 1
Driver mutations	*JAK2*V617F AB > 35% *CALR* type 1 (-like) *MPL*	*JAK2*V617F AB > 50% *JAK2*V617F homozygosity
Myeloid gene variants	*EZH2*, *IDH1/2*, *SF3B1*, *SH2B3*, *TP53*, *U2AF1*	*ASXL1*, *IDH1/2*, *SF3B1*, *SRSF2,* splicing-related
Treatment		Cytopenias/inefficacy on splenomegaly under HU
Biological	MCP-1 polymorphisms NF-kB signaling	MCP-1 polymorphisms NF-kB signaling

Legend: *ET*, essential thrombocythemia; *PV*, polycythemia vera; *pre-PMF*, prefibrotic primary myelofibrosis; *BMF*, bone marrow fibrosis; *MK*, megakaryocytes; *AB*, allele burden; *HU*, hydroxyurea; *MCP-1*, *monocyte chemoattractant protein-1*; *NF-kB*, *nuclear factor k-light-chain-enhancer of activated b cells*

Mora et al. addressed the variability of clinical phenotype and genotype at the time of SMF diagnosis in relation to the TTP from ET/PV [4]. Only in PPV-MF cases, there was a correlation between TTP and lower hemoglobin (Hb) values at SMF evolution [4]. Besides, a significant association between TTP and larger spleen size was found [4]. Looking at driver mutations, genotype was overall associated to TTP [4]: in a Cox regression model that considered age, spleen size, and Hb level at SMF diagnosis, patients with *CALR*-mutated ET had a significantly longer TTP than those with *JAK2*-mutated ET/PV and TN cases [4]. In details, median TTP was 12.1 years (range, 0.4–34.8) for *CALR*-mutated ET, with no imbalance based on mutation subtypes [4]. At the opposite, TN subjects experienced the shortest TTP, which corresponded to 8.2 years (range, 1.8–18.4) [4].

These findings suggest monitoring patients for the development of anemia and/or splenomegaly [4]. Apart from the above evidence, in clinical practice, it could be useful to perform a bone marrow examination in ET and PV patients that develop anyone of the minor criteria for SMF diagnosis to recognize it earlier [3]. Besides, driver mutation signature in ET and *JAK2*V617F AB in PV could be used to establish a genotype-driven follow-up [4, 49, 55]. Information on the predictive role of M-GVs such as splicing mutations is growing, but to date, there is no clear indication for routinely performing NGS in PV/ET and tailoring patients’ monitoring on its results.

## Primary Myelofibrosis

To date, the most widely used prognostic models for PMF are the *International Prognostic Scoring System* (IPSS) [56], applicable at diagnosis, and the *Dynamic IPSS* (DIPSS), at any time during follow-up [12]. These scores share the same clinical variables: age > 65 years, Hb < 10 g/dL, leukocyte count > 25 × 10^9^/L, circulating blasts ≥ 1%, and constitutional symptoms [12, 56]. Every parameter has been given one point, except for anemia in the DIPSS, which weight is two points [12, 56]. OS of the four categories (low, intermediate-1, intermediate-2, and high risk) defined by the IPSS ranges between 11.3 and 2.3 years, while from not reached to 1.5 years in the DIPSS [12, 56]. In both models, intermediate-2- and high-risk groups have an estimated OS below 5 years [12, 56]. Main death reasons in PMF are BP evolution and nonclonal progression [56].

Subsequently, the DIPSS was revised into the DIPSS-plus model [57] that considered also red blood cell (RBC) transfusion need, platelet (PLT) count < 100 × 10^9^/L, and “unfavorable” karyotype [57]. Caramazza et al. identified the latter in complex karyotype (CK) or sole or double abnormalities such as + 8, − 7/7q-, i(17q), inv(3), − 5/5q-, 12p-, or 11q23 rearrangements [58]. IPSS and DIPSS(-plus) are to date the prognostic scores recommended for PMF stratification by the most recent European treatment guidelines [26••].

As for pre-PMF, Guglielmelli et al. have shown that median OS is significantly better compared to overt-PMF cases (14.7 vs 7.2 years) [59•]. Of note, patients with pre-PMF were not included in the development of the overmentioned models, and it was found that IPSS could not discriminated pre-PMF patients well, if their score falls in the intermediate groups [59•].

Since MF is a disease of the elderly, some groups have tried to include also relevant comorbidities in conventional models, but with non-conclusive results [60, 61].

The discoveries related to the molecular background of PMF have led to the investigation of possible correlations between gene alterations and outcome. As for driver mutations, presence of *CALR* type 1 has been associated with favorable prognosis compared to others [59•, 62]. More than 80% of patients with PMF harbor M-GVs [63]. Abnormalities in *ASXL1* (found in around 30% of patients) and less frequent alterations in *SRSF2*, *EZH2*, and *IDH1*/*IDH2* were defined as a high molecular risk (HMR) group, with a prognostic impact proportional to the number of those mutations [59•, 63, 64••]. Therefore, for cases potentially eligible for allo-SCT (aged ≤ 70 years), an integrated *Molecular Enhanced International Prognostic Score System* (MIPSS70) was developed [64••]. Variables included in the MIPSS70 were Hb < 10 g/dL, leukocytes > 25 × 10^9^/L, PLT count < 100 × 10^9^/L, circulating blasts at least 2%, BMF at least grade 2, constitutional symptoms, absence of *CALR* type 1(-like) mutation, presence of HMR mutations, and of two or more HMR alterations [64••]. Median OS was 27.7, 7.1, and 2.3 years in low- (0–1 points), intermediate- (2–4 points), and high (at least 5 points)-risk patients [64••].

The MIPSS70-plus considered the same list of mutations but only three clinical risk factors: Hb < 10 g/dL, circulating blasts ≥ 2%, and constitutional symptoms [64••]. In addition, it included a two-tiered cytogenetic risk variable (unfavorable vs favorable) [64••]. Here, unfavorable karyotype was defined by any AK other than normal karyotype (NK, present in 55% of PMF cases) or sole abnormalities of 20q-, 13q-, + 9, chromosome 1 translocation/duplication, -Y, or sex chromosome abnormality excluding -Y [64••]. The MIPSS70-plus distinguished patients in four different risk categories, with median OS ranging between 20 and 1.7 years in the training cohort [64••]. A further revision (MIPSS70-plus v2.0) incorporated the *U2AF1*Q157 variant as an additional HMR mutation, sex- and severity-adjusted anemia thresholds, and a so-called “very high” cytogenetic risk group, represented by cases with single/multiple abnormalities of − 7, i(17q), inv(3)/3q21, 12p-/12p11.2, 11q-/11q23, or other autosomal trisomies not including + 8/ + 9 (i.e., + 21, + 19) [65••]. Five MIPSS70-plus v2.0 categories were created, with 10-year OS ranging from 92 to less than 5% [65••]. The inclusion of molecular and karyotype information proper of MIPSS70 and MIPSS70-plus v2.0 allows an upstaging of patients who would conventionally be considered to have a favorable prognosis [64••, 65••]. In clinical practice, the high frequency of “dry tap” in PMF limits the use of models based on cytogenetic data, although this information can be obtained from the peripheral blood, as well. In the most recent NCCN (*National Comprehensive Cancer Network*) guidelines, MIPSS70 and MIPSS70-plus v2.0 have been included together with IPSS and DIPSS(-plus) [27••].

The *Genetically Inspired Prognostic Scoring System* (GIPSS) is exclusively based on molecular (absence of *CALR* type 1[-like] mutations, presence of *ASXL1*, *SRSF2* and *U2AF1*Q157) and cytogenetic variables [66••]. Median OS in the derived four risk categories varies from 26.4 to 2 years [66••]. Predictive accuracy of GIPSS was suggested to be comparable to that of MIPSS70-plus [66••]. Table 3 describes the parameters considered in molecularly imprinted PMF prognostic models [64••, 65••, 66••].

**Table 3:** Molecularly based prognostic models for primary myelofibrosis

	MIPSS70	MIPSS70-plus v2.0	GIPSS
Genetic variables (points)	No *CALR* type 1[-like] (1)	No *CALR* type 1[-like] (2)	No *CALR* type 1[-like] (1)
	1 HMR (1)	1 HMR included *U2AF1*Q157 (2)	*ASXL1* (1)
	> 1 HMR (2)	> 1 HMR included *U2AF1*Q157 (3)	*SRSF2* (1)
			*U2AF1*Q157 (1)
		VHR karyotype (4)	VHR karyotype (2)
		UF karyotype (3)	UF karyotype (1)
Clinical variables (points)	Hb < 10 g/dL (1)	Severe anemia (2)	
		Moderate anemia (1)	
	WBC > 25 × 10^9^/l (2)		
	PLT < 100 × 10^9^/l (2)		
	Blasts ≥ 2% (1)	Blasts ≥ 2% (1)	
	Constitutional symptoms (1)	Constitutional symptoms (2)	
	BMF grade ≥ 2 (1)		

Legend: *MIPSS70*, *Molecular Enhanced International Prognostic Score System*; *GIPSS*, *Genetically Inspired Prognostic Scoring System*; *HMR*, high molecular risk; *VHR*, very high risk (single/multiple abnormalities of -7,i(17q),inv(3)/3q21,12p-/12p11.2,11q-/11q23, + 21, or other autosomal trisomies except + 8/9); *UF*, unfavorable (chromosomal abnormalities except VHR or sole 13q-, + 9,20q-, chromosome 1 translocation/duplication or sex chromosome alterations including -Y); *Hb*, hemoglobin; *WBC*, white blood cells; *PLT*, platelets; *BMF*, bone marrow fibrosis

In most of the abovementioned PMF prognostic scores, a widely used variable is circulating blasts count [12, 56, 57, 64••, 65••]. Nevertheless, their definition by morphology is poorly standardizable. A recent paper has investigated the use of multiparameter flow cytometry (MFC) for circulating CD34 + cells count in a small cohort of PMF cases [67]. The derived MFC-enhanced MIPSS70-plus model outperformed its standard counterpart in PMF, opening the possibility of a re-evaluation of the role of MFC, an easily accessible and standardized test, to improve prognostic definition in this disease [67].

Luque Paz et al. have questioned the value of *ASXL1* mutations in MF and proposed a novel model, named “NGS,” that considers four genetic groups [68•]: *TP53* mutated, “High risk” (≥ 1 mutation in *EZH2*, *CBL*, *U2AF1*, *SRSF2*, *IDH1*, and *IDH2*), *ASXL1* mutated-only, and “Others” [68•]. In this study, *ASXL1* abnormalities had a negative prognostic value in MF only when associated with *TP53* or “High risk” genes [68•]. Then, the Florence group has reclassified 330 PMF cases based on this NGS model [69•]: the *TP53* mutated and the “High risk” patients actually showed the worst OS, but the *ASXL1* mutated-only group had a clearly inferior outcome compared to the “Others” [69•]. Among “High risk” PMF patients, *ASXL1* mutations were found in two-thirds of cases and implied a worse outcome [69•].

The association of some molecular alterations with the outcome of certain MF subtypes has been recently noted [70, 71]. RAS/MAPK pathway genes have an unfavorable role on survival only in overt-PMF, even in a multivariate analysis that considered conventional prognostic parameters [70]. In an abstract presented at the 2021 *American Society of Hematology* (ASH) meeting, patients with “myelodepletive” phenotype (at least one among leukocytes < 4 × 10^9^/L, Hb < 11 g/dL for males and < 10 g/dL for females, PLT < 100 × 10^9^/L) presented more frequently TN signature and *ASXL1*, *IDH1/2*, *N/KRAS*, *U2AF1*, and *CUX1* mutations [71]. On univariate analysis, OS was significantly shorter in this subgroup [71].

Reported clinical risk factors for BP transformation in PMF include thrombocytopenia, excess of circulating blasts, marked leukocytosis, RBC transfusion-requiring anemia, and older age [72]. As for chromosomal alterations, particularly detrimental is the role of monosomal karyotype (MK) [73]. From a biological point of view, increased levels of serum interleukin-8 and of C-reactive protein could be involved [72]. Modification of the DIPSS during follow-up may also predict different risks of BP: patients belonging to the higher categories should therefore more strictly monitored for signs of clonal evolution [12, 74]. TN status, HMR mutations, and alterations in RAS/MAPK pathway genes, *RUNX1*, *CEBPA*, or *SH2B3*, have been associated with higher incidence of BP [62, 63, 70, 75]. Since the identified role of HMR mutations in this setting, MIPSS70(-plus) score could also predict BP transformation [64••].

## Secondary Myelofibrosis

At 15 years of follow-up, the cumulative incidence of SMF is equal to 13.4% in *CALR*-mutated ET, 8.4% in *JAK2*-mutated ET, and 13.6% in PV cases [9].

Recent studies have demonstrated that SMF differs from PMF in terms not only of clinical and molecular characteristics, but also of prognosis [76, 77]. As a consequence, specific and detailed information on SMF seemed necessary. In 2014, an international collaboration among 16 countries in Europe and the USA started, called the *MYelofibrosis SECondary to PV and ET* (MYSEC) project [22••]. The original database retrospectively collected 781 PPV- and PET-MF cases [22••].

Within 685 molecularly annotated MYSEC subjects, median OS was 9.3 years for the whole SMF cohort, 14.5 years in PET-MF, and 8.1 years in PPV-MF, with a borderline difference between the two SMF subtypes [22••]. In a multivariable analysis, *CALR*-mutated patients had a better course compared with *JAK2*V617F-mutated PET-MF and PPV-MF [22••].

Since conventional prognostic models developed for PMF patients resulted suboptimal to predict survival in SMF [76, 77], Passamonti et al. applied a Cox regression model to the MYSEC genotype-annotated cases to generate an integrated clinical-molecular prognostic score, called *MYelofibrosis SECondary-Prognostic Model* (MYSEC-PM) [13••]. In details, two points were given to Hb < 11 g/dl, blasts ≥ 3%, and wild-type *CALR*; one point each to PLT count < 150 × 10^9^/L and presence of constitutional symptoms [13••]. Age-related risk was calculated as 0.15 points per year [13••]. Four MYSEC-PM risk categories were created [13••]: low (score < 11), intermediate-1 (11 ≤ score < 14), intermediate-2 (14 ≤ score < 16), and high risk (score ≥ 16) [13••]. Median OS was not reached in the low-risk group, while it was 9.3 years in the intermediate-1-, 4.4 years in the intermediate-2-, and 2 years in the high-risk category [13••]. To help treating physicians in calculating the MYSEC-PM score, a nomogram on the original paper and an online interactive application (available at https://mysec.shinyapps.io/prognostic_model/) have been created [13••].

Differently from PMF, the recently proposed MFC-enhanced MYSEC-PM model did not outperform its standard counterpart [67].

Other prognostic factors have been identified in SMF, thanks to the MYSEC study [78•, 79, 80].

Out of 376 cytogenetic-annotated SMF cases, AK was found in about one third [78•]. Median OS was significantly different between patients with NK and AK (10.1 vs 6.1 years) [78•]. Patients with MK, those with CK without MK, and those with CK had an estimated survival of less than 3.5 years [78•]. Even though the MYSEC-PM outperformed the prognostic relevance of AK in multivariate analysis, the implications of karyotype reinforce the utility of assessing cytogenetics at first suspicion of evolution from PV/ET to SMF [78•]. In another project’s sub analysis, females showed a better outcome compared to males, even adjusting for age at SMF diagnosis [79].

In 2019, the MYSEC database has been enriched with supplemental cases reaching the significant number of 805 SMF [80]. Within this cohort, the prognostic role of BMF grade (2 vs 3) was investigated [80]. The latter was clearly associated with lower OS (7.4 vs 8.2 years) in univariate analysis, claiming for the necessity of an early recognition of evolution from PV/ET to SMF [80].

As for M-GVs, information in SMF is limited. A collaborative Italian study based on NGS methods showed that, among the HMR mutations, only *SRSF2* resulted correlated with reduced OS in PET-MF [81]. Reviewing 193 Institutional SMF cases using the overmentioned “NGS model,” the Florence group showed that *TP53* mutations conferred the worst outcome (median OS 13 months) [68•, 69•]. Differently from PMF, prognosis of cases *ASXL1* mutated-only was not statistically different from the “Others” and the “High risk” category (median OS of 141, 131, and 58 months, respectively) [69•]. Within the latter group, *ASXL1* mutations were found in around two-thirds of cases and did not influence outcome [69•]. RAS/MAPK pathway genes do not seem to play a role in SMF course [70]. A “myelodepletive” phenotype in PPV/PET-MF was found to be associated with *U2AF1* mutations (as in PMF) but also distinctively with *TP53* and *SETBP1* alterations [71]. This subgroup of patients had a significantly shorter OS compared to the counterpart (44 vs 105 months) [71].

NGS analysis of the larger MYSEC cohort is underway and it will shed light on the molecular architecture of this type of MF. The application of artificial intelligence (AI) methods to this wide set of data will lead to the construction of integrated and personalized prognostic scores in SMF [82].

In the MYSEC database, BP incidence resulted significantly higher in *JAK2*V617F and TN vs *CALR-*mutated PET-MF, even adjusting for age at SMF diagnosis [22••]. The topic is currently under investigation within this dataset.

## Special Considerations in Primary and Secondary Myelofibrosis

### The Complexity of Candidates’ Selection to Allogenic Hematopoietic Stem Cell Transplant

As described above, survival could be drastically compromised in MF [12, 13••, 56, 64••]. The most recent European MPNs guidelines (drafted before the implementation of specific scores for SMF in clinical practice) recommend that allo-SCT should be performed in young and fit patients who belong to the most unfavorable risk categories (i.e., in whom the estimated OS is less than five years), as defined by conventional prognostic scores for PMF (IPSS, DIPSS, DIPSS-plus) [26••]. This curative procedure should be also offered to suitable intermediate-1-risk subjects with *ASXL1* mutation [26••]. In the same risk category, Kröger et al. suggested to consider as possible candidates PMF patients either with refractory RBC transfusion–dependent anemia, circulating blasts > 2%, or adverse cytogenetics [83, 84]. The updated NCCN guidelines recommend allo-SCT in PMF for intermediate-2-/high-risk DIPSS(-plus) and in case of score at least 4 by MIPSS70(-plus v2.0), while in SMF for intermediate-2-/high-risk MYSEC-PM cases [27••].

It is anyway getting increasingly evident that the complexity of MF biology and the significant mortality and morbidity rates of allo-SCT require that patients’ selection should be critically made on the integration of more parameters than age and MF prognostic scores [20••, 85••]. Gagelmann et al. recently described a clinical-molecular model (MTSS, *Myelofibrosis Transplant Scoring System*) with the aim of predicting subsequent outcome at the time of referral to allo-SCT [86••]. This model could be applied to both PMF and SMF cases and considers as molecular parameters the presence of *ASXL1* mutation and the absence of *CALR/MPL* [86••]. The other included variables were age ≥ 57 years, Karnofsky performance status lower than 90%, PLT and leukocyte count prior to transplantation (< 150 × 10^9^/L and > 25 × 10^9^/L, respectively), and an HLA (*human leukocyte antigen*)-mismatched unrelated donor [86••]. The latter was assigned two points, as well as wild-type *CALR/MPL* [86••]. All other parameters were given one point [86••]. Patients were therefore clustered in four categories, with a median 5-year OS estimated to be between 90 and 34% [86••]. Mortality from allo-SCT complications varied, inversely, from 10 to 57% in the same time interval [86••]. Based on this study, Passamonti proposed to select for allo-SCT MF patients within 70 years of age, and whose survival is less than 5 years using the most recent disease-specific scores: MIPSS70 for PMF (high-risk category) and MYSEC-PM for SMF (intermediate-2 and high risk) [85••]. Besides, young PMF patients with DIPSS intermediate-1 and mutated for *ASXL1* may be considered [85••]. Then, the MTSS should be applied, in order to identify subjects with better probability of survival and reduced risk complications after allo-SCT [84, 85••]. Low- and intermediate-risk MTSS patients have a clear indication for allo-SCT, while very high risk and high risk aged above 60 years should be reasonably deferred from the procedure in favor of clinical trials [85••].

If these indications are a first step to personalize the allocation to allo-SCT, one should note that in PMF, *ASXL1*-mutated intermediate-1 DIPSS represents a heterogeneous prognostic group and that, in SMF, we are currently looking at prognostic relevance of M-GVs. In both diseases, a better stratification will probably derive from the application of AI methods and the development of different clinical-genomic subgroups [82].

As highlighted in recent publications, many factors inherent to the allo-SCT procedure may influence its outcome, but their description is beyond the scope of this review [87•, 88•].

### Data on Overall Survival with Ruxolitinib

RUX is the first JAK1/2 inhibitor that received approval for MF treatment based on the results of the registrational COMFORT-I/II studies [89, 90]. The latter were not powered to determine the impact of drug on outcome, but—in a data pooled analysis—patients treated with RUX demonstrated significantly improved OS (5.3 vs 3.8 years) compared with patients in the placebo/best available therapy arm, with a reduction in death risk equal to 30% [18••]. Of note, intermediate-2-risk category cases had a clearer benefit with respect to high-risk ones [18••]. This OS improvement was even greater (5.3 vs 2.4 years) when considering censor at cross-over, suggesting the advantages of earlier intervention [18••]. Based on the same pooled data, it has been recently shown that OS at week 240 was significantly improved (63% vs 57%) among patients who initiated RUX at ≤ 12 vs > 12 months from diagnosis [91]. In an ad hoc statistical analysis with a proper patients-matching, Passamonti et al. compared the OS of 100 PMF patients receiving RUX within the COMFORT-II trial with that of 350 DIPSS cases [92]. The former had a significant better OS (5 vs 3.5 years) compared to the seconds, suggesting a potential disease-modifying effect of the drug [92].

Data on OS advantage with RUX are emerging also from real-world (RW) studies [15•, 16•, 93••].

Among those, a recent retrospective analysis on 1677 PMF and SMF patients of the Medicare database showed that median OS was 13.2 months, 44.4 months and not reached before RUX approval, after approval but if RUX-unexposed and in RUX-exposed cases, respectively [15•]. Out of 1010 MF (58% PMF) patients of the ERNEST (*European Registry for Myeloproliferative Neoplasms: Toward a Better Understanding of Epidemiology, Survival, and Treatment*) project, median OS was significantly longer in patients treated with RUX compared with those who received HU (6.7 vs 5.1 years), at a median follow-up of around 5 years [93••]. This difference was even more evident in a propensity score-matching analysis, even though that was performed on a small subgroup [93••].

Several papers have investigated factors impacting OS in RUX-treated patients, both in clinical and in RW settings [94–96, 97••, 98–102, 103•, 104, 105]. Spleen response was identified as predictive of better outcome in a pooled analysis of the COMFORT-I/II trials and in a multicenter Italian study [94, 95]. Looking at CBC, in the registrational trials, the development of anemia in the first 12 weeks of therapy (one of the most common RUX toxicity) did not seem to have a detrimental impact [96]. On this point, more recent data coming from RW (discussed below) provided different results [97••]. Relevant is the number of circulating blasts, as recently reported by Palandri et al. [98]: out of 794 MF cases, median OS was 6.4, 5.7, and 2.5 years in patients with baseline blasts equal to 0%, 1–4%, and 5–9%, respectively [98]. In multivariate analysis, blasts 1–4%, age ≥ 65 years, and the presence of at least two HMR mutations remained significantly associated with a lower survival [98]. In a study by Masarova et al., the presence of at least 10% bone marrow blasts undid the potential benefit of RUX on outcome [99]. In a RW multinational cohort of 469 MF patients including intermediate-1-risk cases and followed for around 34 months, the estimated median OS from RUX initiation was 44.4 months [100]. Factors that negatively impacted prognosis were identified in age ≥ 65 years, PLT count ≤ 200 × 10^9^/L, higher risk categories, comorbidities/performance status, and severe splenomegaly [100]. From a molecular point of view, evidence differs among the various studies: Patel et al. found that the presence of *ASXL1*, *EZH2*, or *IDH1/2* mutations or of at least three M-GVs led to reduced OS [101]. In a targeted deep sequencing analysis of 100 MF patients treated by RUX (77%) or by momelotinib, the unfavorable prognostic role of *ASXL1/EZH2* mutations was confirmed, together with baseline RBC transfusion dependence and high DIPSS risk score [102].

Unfortunately, most patients eventually become resistant or intolerant to RUX, with demonstrated consequent impaired outcome [103•]. Parameters helpful for early identification of such patients, that might benefit from a prompt treatment shift, are lacking. Our group has recently investigated predictors of OS collected after 6 months of RUX in 209 MF patients participating in the RW ambispective observational Italian RUXOREL (*Rete Ematologica Lombarda*)-MF study [97••]. Multivariable analysis identified the following risk factors: RUX dose < 20 mg twice daily at baseline, months 3 and 6 (1 point); palpable spleen length reduction from baseline ≤ 30% at months 3 and 6 (1.5 point); RBC transfusion need at months 3 and/or 6 (1 point); and RBC transfusion need at all time points (i.e., baseline and months 3 and 6—1.5 points) [97••]. A prognostic model, collecting baseline, 3-month and 6-month information, named *Response to Ruxolitinib After 6 Months* (RR6), was developed and dissected three risk categories with different survivals after 6 months of RUX treatment [97••]: low (0 points, median OS not reached, 19% of patients), intermediate (1–2 points, median OS 61 months, 45% of cases), and high (≥ 2.5 points, median OS 33 months, 36% of subjects) [97••]. This model is a proposal and needs further validation, but it could be useful for early shifting of selected intermediate- and high-risk patients to second-line therapies, as investigational trials or even allo-SCT [97••]. A web-based calculator is available to help treating clinicians to define patient’s RR6 score (http://www.rr6.eu/) [97••].

### Recent Biological Insights

An interesting research field in MPNs is the prognostic role of gene expression (GE) signatures [104]. Evaluating the expression of 201 genes in MF circulating granulocytes, outcome-related transcripts were identified and used to differentiate two groups of patients [104]. The so-called “high-risk” subjects displayed an inferior OS and BP-FS compared with “low-risk” cases [104]. The latter were enriched in pre-PMF patients, whereas higher percentages of PPV/PET-MF were present in the high-risk category [104]. The GE-based classification showed quite good agreement with contemporary MF prognostic models and the authors suggested that it could improve the survival prediction of the conventional intermediate-risk groups, with possible redefinition of treatment strategies [104].

Long non-coding RNAs (lncRNAs) have been recently proposed as biomarkers for cancer diagnosis and prognosis [105]. In plasma samples of 41 PMF and 42 SMF cases, Fantini et al. have demonstrated the increased expression profile of a set of circulating lncRNAs, among which LINC01268 level resulted associated with reduced OS and BP-FS, when considering DIPSS classification [105].

Reactive oxygen species (ROS) are an essential component of inflammation-induced oxidative damage to cellular components including DNA, therefore leading to oxidative stress and genomic instability [106]. In CD34 + hematopoietic stem/progenitor cells derived from *JAK2*V617F-mutated PMF and SMF, high plasma levels of total antioxidant capacity showed a correlation with shorter OS, also in multivariate analysis [106].

## Conclusions

The increased knowledge about the pathogenesis and the molecular biology of MPNs has broadened and improved their prognostic definition in recent years. The inclusion of molecular data in survival models is now well established and recommended in PMF, while in patients with PV and ET, the evidence is still preliminary. For the latter two, subjects’ monitoring is still based more on thrombotic risk than on mortality estimates. The evidence that SMF is a different entity compared to PMF has led to a greater alertness in recognizing possible signs of evolution from a pre-existing PV/ET and to the definition of an ad hoc prognostic score, the MYSEC-PM. NGS analysis results of the MYSEC database will definitively shed light on the biological architecture of SMF, opening the way to integrated models for survival stratification also in this disease. Transformation to BP is accompanied by high mortality, and unfortunately at present conventional MPNs prognostic scores cannot accurately predict the risk of this evolution.

Despite an earlier diagnosis, an increase in available therapies (such as RUX), and more experience in the management of allo-SCT, the prognosis of patients with PMF and SMF still remains the real sore point in MPNs field. In the transplant setting, the application of the MTSS to patients selected as having an unfavorable risk by disease-specific scores allows to predict in a personalized way who will have the best outcome and the lowest risk of allo-SCT complications. For the larger cohort of subjects not eligible for curative therapy and treated with RUX, an unmet clinical need is the definition of parameters that are associated with reduced survival. In this context, the RR6 model could represent a useful tool to early shifting patients to novel treatments. As the development of new agents in MF is increasing and a rapidly evolving field, response to first-line therapies might represent a possible endpoint for patients’ stratification [107].
